# Characteristics that increase the risk for pain on propofol injection

**DOI:** 10.1186/s12871-024-02573-y

**Published:** 2024-05-28

**Authors:** Phillip J. Leff, Brett A. Dinner, Keng-Yu Chuang, David B. Leff

**Affiliations:** 1 Department of Internal Medicine, Creighton University Phoenix, 350 W Thomas Rd, Phoenix, AZ 85013 USA; 2 Department of Gastroenterology, Creighton University Phoenix, 350 W Thomas Rd, Phoenix, AZ 85013 USA; 3 Central Arizona Medical Associates, 3638 E Southern Ave, Ste C108, Mesa, AZ USA

**Keywords:** Propofol, Pain on propofol injection, POPI

## Abstract

**Background:**

Propofol for anesthesia has become increasingly popular for endoscopic procedures. However, pain on propofol injection (POPI) remains an issue with administration. The primary endpoint of this study was to identify patient characteristics and factors, such as IV site and gauge, that could predict the occurrence of POPI.

**Methods:**

This was a prospective chart review study of 291 patients undergoing endoscopic procedures. The patient’s demographics, intravenous (IV) site, and gauge were extrapolated. POPI was scored 0–3: 0 for no pain, 1 for minimal discomfort or awareness of sensation, 2 for discomfort but manageable/tolerable, and 3 for severe discomfort with writhing.

**Results:**

291 patient charts were reviewed. One patient was excluded for a lower extremity IV site. 225 (77.6%) had no pain, 48 (16.6%) grade 1 pain, 16 (5.5%) grade 2 pain, and 1 (0.3%) grade 3 pain. 137, 13, and 140 patients respectively had antecubital (AC), forearm, and hand IVs. Zero patients with an AC IV experienced a score greater than 1. Compared to AC, forearm IVs with pain of 2–3 had a univariate odds ratio (OR) of 11.3 (0.66,1.92; *p*-value < 0.001), and hand IVs had a univariate OR of 18.8 (2.46,143.3; *p*-value < 0.001) with a multivariable OR 15.2 (1.93,118.9; *p*-value 0.004). Patients with anxiety/depression and pain had a univariate OR 2.31 (1.09, 7.27; *p*-value 0.031) with a multivariable OR 2.85 (1.06, 7.74; *p*-value 0.039). SSRI/SNRI use had a univariate OR 1.56 (0.57,4.28; *p*-value 0.38). Alcohol use had a univariate OR 1.24 (0.39,3.91; *p*-value 0.71). Narcotic use had a Univariate OR 6.18 (1.49,25.6; *p*-value 0.012). Diabetic patients had a univariate OR of 1.42 (0.45,4.48; *p*-value 0.55). Chronic pain had a univariate OR of 3.11 (1.04,9.28; *p*-value 0.042). Females had a univariate OR 0.98 (0.37,2.63; *p*-value 0.95).

**Conclusion:**

This study identified potential characteristics for having POPI. The incidence of POPI was statistically significant in patients with hand and forearm IVs compared to AC IV sites, larger IV gauges, history of depression/anxiety, history of chronic narcotic use, fibromyalgia, and chronic pain syndromes. This shows the potential of premedicating with analgesics or using AC sites on these select patients to help reduce the risk of POPI.

## Background

Propofol for anesthesia is popular for routine endoscopic procedures, however pain on injection remains an issue. Propofol is the most widely used intravenous (IV) anesthetic agent for induction and maintenance of anesthesia. [1, 2] Although pain on propofol injection (POPI) is transient and patients are often amnestic to it, pain can be considerable. In one study, 91% of patients did not recall pain post-procedure, and only 2.6% reported remembering severe pain. [3] It is estimated that pain occurs in 28–90% of patients. [4–6] Studies to date have postulated assorted reasons why POPI occurs, yet limited evidence exists on the demographics of patients who could be most affected.

Many factors have been suggested to affect the incidence of POPI, including the site of the injection, size of the vein, rapidity of the propofol injection, and the concentration of the propofol. [7] One factor that is often identified in the literature is the peripheral IV (PIV) site. Multiple studies have shown minimal to no pain associated with POPI when propofol is injected into the antecubital (AC) vein as compared to hand IVs. [6–10] Another factor studied was time of exposure to the vein wall in which slow injection caused more pain than rapid bolus. [9] Klement and Arndt suggested that the painful sensation from veins originates from neural elements within the vein wall by way of free afferent nerve endings between the media and the intima. [11] It has been postulated that pain is the result of an indirect irritant effect via the release of kininogen from the kinin cascade. This produces bradykinin, causing vasodilation and hyper-permeability, which may increase the contact between the Propofol phenol group and nerve endings which stimulates pain. [8, 9]

The goals of this study were to understand POPI frequency and identify patient characteristics and factors such as IV site and gauge which could be predictive of POPI. We examined anxiety and depression, selective-serotonin reuptake inhibitors (SSRI)/ Serotonin-Norepinephrine Reuptake Inhibitors (SNRI) use, chronic pain, narcotic use, alcohol use, fibromyalgia, and gender to determine any discriminating factors.

## Methods

A prospective chart review study was conducted analyzing consecutive patients within a preset timeframe of September-December 2022 (*n* = 291) undergoing outpatient endoscopic procedures (colonoscopy and upper endoscopy). Chart review and de-identification were performed by the principal investigator, and further data analysis on de-identified data was performed by sub-investigators. The study was approved by the Valleywise Health Institutional Review Board (IRB), and the requirement for written informed consent was waived by the IRB as the study did not change any patient management and was a chart review study. Collected data was analyzed through RedCap, a web-based application to capture data for clinical research. [13, 14] Strengthening the reporting of observational studies in epidemiology checklist was used for standardization and reproducibility. The procedures were performed at a private urban community endoscopy center with two experienced nurse anesthetists providing propofol sedation from September through December 2022. All patients over the age of 18 receiving propofol during the preset timeframe of the study were included. Patients under the age of 18 or patients with non-upper-extremity IV sites were excluded. One patient had an IV placed in the foot and was excluded from the study. IV site and gauge for a patient were decided by registered nurses (RN) not associated with this study in the pre-operative area at their discretion. ASA score varied from 1 to 3 and the age range was 20–88.

Notated were the patient’s age, gender, peripheral intravenous (PIV) site, and gauge. No patients were given premedication or co-administered medications per standard of practice at this facility. The certified registered nurse anesthetist (CRNA) assessed the amount of discomfort the patient had based on the patient’s observed response. Patients were asked how they felt during the initiation of propofol injection and multiple times over the following 60 s with the highest score used. They were given a score of 0 for no pain, 1 for minimal discomfort or awareness of any sensation at their IV sites, 2 for discomfort but manageable/tolerable, and 3 for severe discomfort with writhing. 10 mg/ml of propofol was injected manually at an approximate rate of 200 mg/minute in all patients regardless of the IV site to levels of appropriate sedation for endoscopy. The total dose of propofol varied based on the patient’s sedation level, and a top-up method was used throughout the procedures. Patients were monitored during procedures with regular blood pressure checks, end-tidal capnography, telemetry, and close aspiration/apnea monitoring. The medical charts were reviewed for history or presence of anxiety/depression, SSRI/SSNI use, diabetes, chronic pain/back pain, narcotic use, and regular alcohol use as defined by more than one drink/day.

Various statistical models were used for data analysis to calculate odds ratios (OR) and *p*-values. Continuous variables were compared using the Kruskal–Wallis Test. Categorical variables were compared using Chi-squared analysis or Fisher’s Exact Test. Univariate Ordinal Logistic Regression with no adjustments (Univariate OLR) and Multivariable Ordinal Logistic Regression following a backward variable selection (Multivariable OLR) were each conducted. Univariate Binary Logistic Regression with no adjustments (Univariate BLR) and Multivariable Binary Logistic Regression following a backward variable selection (Multivariable BLR) were conducted. The *p*-value threshold from univariate analysis for covariates to be included for the backward variable selection was 0.20.

## Results

291 patients’ data was collected, and one patient was excluded due to lower extremity IV placement. 290 patient charts were reviewed; the breakdown of pain was: no pain in 225 patients (77.6%); grade 1 in 48 patients (16.6%); grade 2 in 16 patients (5.5%); grade 3 in 1 patient (0.3%).

Tabulated data analysis can be seen in Tables 1, 2 and 3.

**Table 1:** POPI pain scores analyzed by specific variables. Continuous variables were compared using the Kruskal–Wallis Test (age). Categorical variables were compared using Chi-squared analysis or Fisher’s Exact Test. Percents were obtained through a comparison of specific variables versus the total number within that pain category

POPI Pain Score by Variables					
Variables	Overall	0	1	2 or 3	*p*-value
*N* (%)	290	225 (77.6)	48 (16.5)	17 (5.84)	
Age, years (mean, SD)	60.3 (12.6)	60.5 (12.9)	60.0 (11.8)	59.6 (10.9)	0.85
Sex, female (*n*, %)	155 (53.3)	133 (59.1)	23 (47.9)	9 (52.9)	0.72
IV Site (*n*, %)					< 0.001
AC	137 (47.2)	127 (56.4)	10 (20.8)	0 (0.0)	
Forearm	13 (4.48)	8 (3.56)	4 (8.33)	1 (5.88)	
Hand	140 (48.3)	90 (40.0)	34 (70.8)	16 (94.1)	
IV Gauge (*n*, %)					0.002
20	9 (3.10)	6 (2.65)	3 (6.25)	0 (0.0)	
22	265 (91.4)	213 (94.3)	39 (91.3)	13 (76.5)	
24	17 (5.9)	7 (3.1)	6 (12.5)	4 (23.5)	
Anxiety/Depression (yes,%)	100 (34.4)	73 (32.3)	16 (33.3)	11 (64.7)	0.031
Diabetes (yes,%)	47 (16.2)	32 (14.2)	11 (22.9)	4 (23.5)	0.19
SSRI/SNRI (yes,%)	68 (23.4)	50 (22.1)	12 (25.0)	6 (35.3)	0.40
Narcotics (yes,%)	11 (3.78)	7 (3.10)	1 (2.08)	3 (17.1)	0.035
Alcohol (yes,%)	52 (17.9)	38 (16.8)	11 (22.9)	3 (17.7)	0.57
Fibromyalgia (yes,%)	2 (0.69)	0 (0.0)	1 (2.08)	1 (5.88)	0.023
Chronic Back Pain (yes,%)	33 (11.3)	22 (9.73)	6 (12.5)	5 (29.4)	0.054

**Table 2:** POPI pain score using Univariate Ordinal Logistic Regression with no adjustments. Multivariable Ordinal Logistic Regression following a backward variable selection comparing variables to pain scores 0,1, and 2 or 3. Multivariable regression was performed on univariate values with a *p*-value < 0.2

Ordinal Logistic Regression of POPI by Variables				
Variables	Univariate		Multivariable	
	OR (95% CI)	*P*-trend	OR (95% CI)	*P*-trend
Age, years	0.99 (0.97, 1.02)	0.75		
Sex, female	0.82 (0.47, 1.42)	0.49		
IV Site				
AC	REF	< 0.001	REF	< 0.001
Forearm	7.70 (2.18, 27.2)		5.45 (1.43, 20.7)	
Hand	7.36 (3.55, 15.3)		3.72 (0.72, 19.0)	
IV Gauge				
20	REF	0.002	REF	0.027
22	0.55 (0.14, 2.19)		1.12 (0.27, 4.58)	
24	3.32 (0.66, 16.6)		3.72 (0.73, 19.0)	
Anxiety/Depression	1.60 (0.91, 2.91)	0.10	1.75 (0.95, 3.21)	0.065
Diabetes	1.80 (0.91, 3.55)	0.089		
SSRI/SNRI	1.39 (0.74, 2.58)	0.30		
Narcotics	2.73 (0.75, 9.90)	0.13		
Alcohol	1.32 (0.67, 2.61)	0.42		
Fibromyalgia	22.8 (1.78, 293.1)	0.016		
Chronic Back Pain	2.07 (0.95, 4.51)	0.067

**Table 3:** POPI pain score using Univariate Binary Logistic Regression with no adjustments. Multivariable Binary Logistic Regression following a backward variable selection comparing scores of 0 or 1 to 2 or 3. Multivariable regression was performed on univariate values with a *p*-value < 0.2

Binary Logistic Regression of POPI by Variables						
Variables	Pain Score		Univariate		Multivariable	
	0 or 1	2 or 3	OR (95% CI)	*p*-value	OR (95% CI)	*p*-value
*N* (%)	273 (94.14)	17 (5.86)				
Age, years (mean, SD)	60.4 (12.7)	59.6 (10.9)	0.99 (0.96, 1.03)	0.91		
Sex, female (*n*, %)	146 (53.3)	9 (52.9)	0.98 (0.37, 2.63)	0.95		
IV Site (*n*, %)						
AC	137 (50.2)	0 (0.0)	REF	< 0.001	REF	0.004
Forearm	12 (4.40)	1 (5.88)	11.3 (0.66, 1.92)		6.06 (0.32, 114.8)	
Hand	124 (45.4)	16 (94.1)	18.8 (2.46, 143.3)		15.2 (1.93, 118.9)	
IV Gauge (*n*, %)						
20	9 (3.28)	0 (0.0)	REF	0.007	REF	0.049
22	252 (91.9)	13 (76.5)	0.41 (0.047, 3.55)		0.75 (0.08, 6.81)	
24	13 (4.74)	4 (23.5)	3.33 (0.32, 34.2)		3.46 (0.32, 37.3)	
Anxiety/Depression (yes,%)	89 (32.5)	11 (64.7)	2.31 (1.09, 7.27)	0.031	2.85 (1.06, 7.74)	0.039
Diabetes (yes,%)	43 (15.7)	4 (23.5)	1.42 (0.45, 4.48)	0.55		
SSRI/SNRI (yes,%)	62 (22.6)	6 (35.3)	1.56 (0.57, 4.28)	0.38		
Narcotics (yes,%)	8 (2.92)	3 (17.1)	6.18 (1.49, 25.6)	0.012		
Alcohol (yes,%)	49 (17.9)	3 (17.7)	1.24 (0.39, 3.91)	0.71		
Fibromyalgia (yes,%)	1 (0.36)	1 (5.88)	15.0 (0.90, 250.7)	0.059		
Chronic Back Pain (yes,%)	28 (10.2)	5 (29.4)	3.11 (1.04, 9.28)	0.042		

### IV site

Three PIV sites were used during this investigation: AC, forearm, and hand: 137, 13, and 140 patients, respectively. AC site had 0 patients with a 2–3 pain score (0%). One (7.69%) patient with a forearm IV had a pain score of 2–3, corresponding to a statistically significant confidence interval using ordinal regression, and non-statistically significant confidence intervals on binary analysis. 16 (11.43%) patients with a hand IV site had a pain score of 2–3, corresponding to a statistically significant Univariate BLR OR 18.8 (2.46,143.3; *p*-value < 0.001) and multivariable BLR OR 15.2 (1.93,118.9; *p*-value 0.004) on binary analysis. Association analysis of pain scores by IV site was statistically significant across IV sites.

### IV gauge

Three IV gauges were used during this investigation: 20, 22, and 24. There were 9 (3.1%) patients with a 20 gauge IV, there were 265 (91.4%) patients with a 22 gauge IV, and 17 (5.9%) patients with a 24 gauge IV. 13 (4.9%) patients with a 20, 22, and 24 gauge IVs had a pain score 2–3, corresponding to a Univariate BLR OR and a Multivariable BLR OR confidence interval that was not statistically significant. Association analysis of pain scores by IV gauge was statistically significant.

### Anxiety/depression

100 of the 290 total patients had a documented history of anxiety and/or depression. 11 (11%) patients of those with a documented history of anxiety and/or depression experienced pain > 1 on the pain scale compared to 6 (3.16%) patients of those without such documented history of anxiety and/or depression had pain, corresponding to a univariate BLR OR 2.31(1.09, 7.27; *p*-value 0.031) with a multivariable BLR OR 2.85 (1.06, 7.74; *p*-value 0.039) which are statistically significant. Of the 153 patients who had hand/forearm IVs, 54 patients had a documented history of anxiety and/or depression, while 99 patients were without such documented history. Pain was experienced in 11 (20.4%) patients with a documented history of anxiety and/or depression compared to 6 (6.1%) patients without a documented history. Association analysis of pain scores with and without a history of anxiety and/or depression was statistically significant.

### Alcohol use

52 of the 290 total patients were identified as drinking more than one alcoholic beverage per day. Of alcohol users, 49 patients experienced a pain score of 0 or 1 (94.2%), and 3 patients experienced a pain scale of 2 or 3 (5.8%). There were 238 patients without alcohol use of whom 14 (5.9%) had pain. A univariate BLR OR with a confidence interval that was not statistically significant. Of the 153 patients who had hand/forearm IVs, there were 29 patients with alcohol use, of which 3 (10.3%) had pain, and 124 patients without alcohol use, of which 13 (10.5%) had pain. Association analysis of pain scores with and without alcohol use was not statistically significant.

### Diabetes mellitus

46 of the 290 total patients had a documented history of diabetes compared to 244 patients without diabetes. Four (8.7%) patients with a history of diabetes experienced pain > 1 on the pain score, compared to 14 (5.7%) patients without a history of diabetes had pain. Univariate BLR OR that was not statistically significant. Of the 153 patients, who had hand/forearm IVs, 27 patients had a documented history of diabetes compared to 126 patients without diabetes. Of patients with hand/forearm IVs, (14.8%) of patients with a history of diabetes had pain, and 13 (10.3%) patients without a history of diabetes had pain. Association analysis of pain scores with diabetes was not statistically significant.

### SSRI/SNRI use

68 of the 290 total patients had SSRI/SNRI use, and 6 (8.8%) experienced pain. Of the 222 patients without SSRI/SNRI use, 11 (5.0%) had pain. The univariate BLR OR was not statistically significant. Of the 153 patients who had hand/forearm IVs, there were 41 patients with SSRI/SNRI use and 112 without. 6 (14.6%) patients with SSRI/SNRI use had pain and 11 (9.8%) patients without SSRI/SNRI use had pain. Association analysis of pain scores with and without SSRI/SNRI was not statistically significant.

### Narcotic use

11 of the 290 total patients used narcotics regularly compared to 279 patients without narcotics use. 3 (27.3%) patients with regular narcotic use experienced pain, while 14 (5.0%) patients without regular narcotic use had pain. Univariate BLR OR was statistically significant. Of the 153 patients who had hand/forearm IVs, there were 7 patients with regular narcotic use and 146 without. 3 (42.9%) patients with regular narcotic use had pain and 14 (9.6%) patients without regular narcotic use had pain. Association analysis of pain scores with and without a history of narcotic use was statistically significant.

### Chronic pain/back pain

33 of the 290 total patients had chronic pain issues and 257 did not. 5 (15.2%) patients with chronic pain experienced pain, while 12 (4.7%) patients without chronic pain experienced pain. Univariate BLR OR was statistically significant. Of the 153 patients who had hand/forearm IVs, there were 20 patients with chronic pain issues and 133 patients without. 5 (25%) patients with chronic pain experienced pain12 (9.0%) patients without chronic pain experienced pain. Association analysis of pain scores with and without chronic pain was not statistically significant.

### Age/gender

The average age of participants in the study was 60.3 years. There was no statistical significance seen among age and POPI. 135 of the 290 total patients were male, of whom 8 (5.9%) experienced pain. 155 of the 290 total patients were female, of whom 9 (5.8%) experienced pain > 1 on the pain scale. Univariate BLR OR and chi-squared analysis showed no statistical significance.

### Fibromyalgia

Two of the 290 patients had fibromyalgia, of the two patients, one (50%) had a pain level of 2,3, and one (50%) patient had a pain level of 1. Fibromyalgia had a univariate OLR of 22.8 (1.78,293.1; *p*-value 0.016). Fibromyalgia was statistically significant with Fisher’s exact test.

## Discussion

Historically POPI has been considered significant enough that many anesthesia providers will prophylactically use lidocaine or other modalities such as narcotics, ketamine, blood aspiration, or tourniquet use to avert pain. [12] Studies have assessed how to alleviate POPI with lidocaine, decreasing the speed of injection, dilution, ketamine, narcotics, use of a tourniquet, etc. [12]

Our study identified that there is a significant amount of POPI of upwards to 38%, yet this is limited to small hand/forearm IVs, which was statistically significant. The prevalence of pain was approximately equivalent for IV placement in the forearm and hand (35.7% vs 38%), suggesting that these are smaller IVs compared to those placed in the larger AC vein and that the size of the vein may be significant in predicting pain**.** The incidence of pain in AC veins was only 8%, and all those occurrences were of minimal significance. No one scored greater than a grade 1, which was considered casual awareness of injection into the vein without any significant pain. As such, we would recommend that lidocaine or other prophylactic modalities not be used with AC IVs. The preference of our institution, however, is to place hand IVs for convenience and accessibility for the anesthesia provider as well as for the lower risk of infiltration and phlebitis. [34] No significant difference in POPI was apparent across genders. Of the 17 patients that had a grade 2 or 3 pain reaction, 88% had possible predictive characteristics. Notably, only 11% of patients with small vein IVs had significant (grade 2 or 3) pain; the vast majority were grade 1 pain (27%). POPI by IV gauge was statistically significant. POPI by IV gauge may be indirectly related to the IV site.

POPI was statistically significant for a history of anxiety and/or depression yet not for the use of SSRI/SNRI, the first-line medical treatment of depression and anxiety. Further data collection and analysis need to be conducted to better understand this discrepancy.

Chi-squared analysis and Fisher’s Exact Tests found statistical significance in pain scores across IV sites, IV gauge, anxiety/depression, narcotics, and fibromyalgia. Univariate Ordinal Logistic Regression with no adjustments and Multivariable Ordinal Logistic Regression following a backward variable selection models found statistical significance across IV sites. Univariate Binary Logistic Regression with no adjustments and Multivariable Binary Logistic Regression following a backward variable selection found statistical significance in pain score across IV sites, anxiety/depression, narcotics, and chronic back pain.

As a result, for patients with IVs in the hand/forearm, small IVs, and a medical history of depression/anxiety, chronic narcotic use, fibromyalgia, and chronic pain syndromes, pain prophylaxis may be beneficial for reducing the incidence of POPI.

Several studies have been performed investigating strategies to alleviate POPI, and these have included the site of injection, premedication with local anesthetics, opiates and ketamine, and aspiration of blood. The most commonly used technique currently is lidocaine. [7, 15–22] It is presumed that this provides a local anesthetic effect on the vein. Studies have shown that administration of local anesthetic immediately before propofol reduced the incidence of pain respectively from 37 to 17%, 49% to 21%, and 64% to 44% when using hand veins. [23–25] Manger and Holak found that administering lidocaine 100 mg one minute before propofol injection reduced the severity but not the incidence of pain. [5] In contrast, lidocaine 100 mg administered after an arm tourniquet was inflated to 50 mmHg for one minute virtually abolished the POPI. [26–28] Several studies have investigated the use of opiates, such as fentanyl. Bahar et al. found that 0.1 mg of fentanyl three to five minutes before propofol injection reduced pain severity but not the overall pain incidence. [29] Helmers et al. found a significant reduction in the incidence of propofol injection pain from 40 to 16% with fentanyl. [30] Ketamine studies found that pretreatment with ketamine 10 mg 30 s before propofol injection significantly reduced the incidence of pain from 84 to 26%. [31, 32] A study by McDonald and Jamison examined the aspiration of 2 ml of the patient’s blood into the syringe of propofol immediately before injection reduced the incidence of pain. [33]

Limitations and considerations for this study include that this was not a randomized study. There is not an even distribution of patients in each IV site and gauge, as the preferred site and gauge from the RNs placing the IVs were used. This could potentially skew results and should attempt to be accounted for in any future studies. Other limitations are that different rates of injection were not examined, or accounted for other factors such as medications, BMI, and OSA. Our chart analysis did not allow for the separation of anxiety/depression and would recommend a repeat study analyzing these disease processes as separate entities to analyze variation. We recommend that our study be repeated with larger sample sizes to further explore the association of POPI with the variables tested. This study is important to correlate patients who would benefit from pain aversion treatment options for those patients identified at high risk, including those with hand/forearm IVs with a history of depression/anxiety, chronic narcotic use, or chronic pain syndromes. Prophylactic use of lidocaine, fentanyl, ketamine, tourniquet, or blood aspiration are reasonable treatment options.

## Conclusions

Patients with an IV in the hand/forearm, and a medical history of depression/anxiety, chronic narcotic use, fibromyalgia, and chronic pain syndromes had statistically significant more POPI. The use of pain prophylaxis may be beneficial in this subset of patients to reduce the incidence of POPI.

## Data Availability

The datasets used and/or analyzed during the current study are available from the corresponding author upon reasonable request.

## Abbreviations

IV: Intravenous
POPI: Pain on propofol injection
PIV: Peripheral IV
AC: Antecubital
SSRI: Selective-serotonin reuptake inhibitors
SNRI: Serotonin-norepinephrine reuptake inhibitors
IRB: Institutional review board
OR: Odds ratio
OLR: Ordinal logistic regression with no adjustments
BLR: Binary logistic regression with no adjustments
